# Prevalence and Patterns of Permanent Tooth Agenesis in Patients With Crouzon or Apert Syndrome: A Systematic Review and Meta‐Analysis

**DOI:** 10.1111/ocr.70046

**Published:** 2025-10-14

**Authors:** M. Cecilia Becerril Santos, Edwin M. Ongkosuwito, Alexandra K. Papadopoulou, Gregory S. Antonarakis

**Affiliations:** ^1^ Division of Orthodontics University Clinics of Dental Medicine, University of Geneva Geneva Switzerland; ^2^ Section of Orthodontics and Craniofacial Biology, Department of Dentistry Radboud University Medical Center Nijmegen the Netherlands

**Keywords:** Apert syndrome, Crouzon syndrome, hypodontia, meta‐analysis, tooth agenesis

## Abstract

Crouzon and Apert syndromes are rare syndromic craniosynostoses frequently associated with craniofacial and dental anomalies, including tooth agenesis. Although individual studies have reported tooth agenesis prevalence data in specific populations, no attempts have been made to systematically synthesise these data. This systematic review and meta‐analysis evaluated the prevalence and patterns of permanent tooth agenesis in patients with Crouzon or Apert syndromes. The study followed a pre‐registered protocol (PROSPERO CRD42024581856). Two independent investigators conducted a literature search in MEDLINE (via PubMed), Web of Science, Embase, ProQuest, and Google Scholar to identify studies on permanent tooth agenesis in individuals with Crouzon or Apert syndromes. Studies were deemed eligible if they reported on permanent tooth agenesis in patients diagnosed with Crouzon or Apert syndromes. Risk of bias was assessed using the Joanna Briggs Institute tool. Random‐effects and inverse variance heterogeneity meta‐analysis models were used for data synthesis. After database search, deduplication, and screening, seven studies were included, comprising a total of 89 individuals with Apert and 77 with Crouzon syndrome. The estimated overall prevalence of permanent tooth agenesis, excluding third molars, was 37% (95% CI: 28%–48%) in individuals with Apert and 31% (95% CI: 17%–46%) in those with Crouzon syndrome. In Apert syndrome, the most common tooth agenesis pattern was the bilateral absence of the mandibular second premolars, followed by the absence of the left maxillary lateral incisors and the bilateral absence of the maxillary lateral incisors. In Crouzon syndrome, the most frequent tooth agenesis pattern was the absence of the right mandibular second premolar, followed by the bilateral absence of the mandibular second premolars. Individuals with Crouzon or Apert syndrome present a high prevalence of tooth agenesis. Early diagnosis of missing permanent teeth is essential to optimise long‐term treatment planning and improve functional and aesthetic outcomes. However, the evidence is limited by small sample sizes and heterogeneity across included studies.

## 1 Introduction

Tooth agenesis in the form of congenitally missing teeth is the most common dental anomaly in the general population, with its prevalence ranging from 3.2%–7.6% (excluding third molars) [1]. The prevalence of permanent tooth agenesis varies depending on the affected teeth, with the third molars being the most commonly absent teeth, with a prevalence of about 20%. This is followed by the mandibular second premolars (2.9%–3.2%), maxillary lateral incisors (1.6%–1.8%), and maxillary second premolars (1.4%–1.6%). A single tooth is missing in around 50% of the patients affected by tooth agenesis, while the absence of two teeth affects one‐third of patients with permanent tooth agenesis [1].

Permanent tooth agenesis impacts various aspects of a patient's life. It may lead to malocclusions that can have psychological implications on patients' appearance, perception, and self‐esteem, while it can also negatively influence functional parameters such as mastication and subsequent digestion, phonetics, and aesthetics. Socially, it may create difficulties for eating and drinking [2], and limit personal and professional development and hamper communication [3, 4].

The aetiopathogenesis of tooth agenesis has been attributed to genetic mutations interfering with the developmental pathways that regulate the complex epithelial‐mesenchymal interaction during embryogenesis [4, 5]. More specifically, mutations in genes such as muscle segment homeobox 1 (*MSX1*), paired box 9 (*PAX9*), fibroblast growth factor receptor 2 (*FGFR2*), axin inhibition protein 2 (*AXIN2*), or sonic hedgehog (SHH) may result in abnormal tooth formation at both the morphological and cellular levels. These genes play key roles in mediating epithelial‐mesenchymal cell interactions and fibroblast growth factor activity, both of which are essential for the synthesis of fibrous material and extracellular matrix critical for proper dental development [4, 5, 6, 7, 8].

Tooth agenesis can occur as an isolated non‐syndromic condition or in the context of an associated syndrome. There are numerous syndromes and conditions that present with tooth agenesis as part of their clinical phenotype, including Down syndrome, Robin sequence, ectodermal dysplasia, and syndromic craniosynostosis [9, 10, 11]. Craniosynostosis is the premature fusion of one or more cranial sutures, restricting skull growth and leading to cranial dysmorphia, cranial base hypoplasia, and maxillofacial deformities [12, 13, 14]. The prevalence of craniosynostosis is estimated at 3.1–7.2 per 10,000 live births, with syndromes such as Apert and Crouzon accounting for 4.5% and 4.8% of all craniosynostosis cases, respectively [12, 13, 14].

Apert syndrome, also known as acrocephaly, has a prevalence of 1 in 80,000–200,000 live births and primarily involves premature fusion of the coronal suture, resulting in a characteristic cone‐shaped cranial growth from the glabella to the posterior fontanelle. This cranial feature, along with facial anomalies and syndactyly of the hands and feet, forms the triad of Apert syndrome, often leading to motor impairments [14, 15]. Additionally, patients with Apert syndrome may experience fusion of the cervical vertebrae (C5–C6), hearing impairments, acne, excessive sweating, respiratory problems such as obstructive sleep apnoea, mouth breathing, and, in some cases, cognitive impairments [12, 13, 14].

On the other hand, Crouzon syndrome with a prevalence of about 1 in 60,000 live births is characterised by premature fusion of all cranial sutures, resulting in a broad skull. These patients may also present with cervical vertebral fusion (C2–C3) and a beak‐shaped nose [14, 15].

Both syndromes share overlapping features, including facial asymmetry, a flat face with a short cranial fossa, hypertelorism, bulging orbits with proptosis, a depressed nasal bridge, a short upper lip, and midface retrusion leading to maxillary hypoplasia and relative mandibular prognathism [12, 13, 14, 15, 16]. These conditions are autosomal dominant in their mode of transmission and are caused by mutations in *FGFR* genes. In Apert syndrome, the mutation occurs in the *FGFR2* gene on the long arm of chromosome 10 [15], while in Crouzon syndrome, the mutation is found in the *FGFR3* gene on the short arm of chromosome 4 [14].

Due to the rarity of Apert and Crouzon syndromes, there is limited evidence in the existing literature regarding the prevalence and patterns of permanent tooth agenesis, as existing studies are based on small samples, in specific populations, with heterogeneous results. The present systematic review and meta‐analysis aimed to investigate in a systematic manner the available evidence by synthesising data from multiple studies, analysing pooled samples of patients and evaluating the prevalence and patterns of permanent tooth agenesis in individuals with Apert or Crouzon syndrome.

## 2 Methods

The reporting of the present systematic review and meta‐analysis was based on the PRISMA (Preferred Reporting Items for Systematic reviews and Meta‐Analyses) 2020 guidelines [17]. The protocol was registered a priori in PROSPERO (CRD42024581856).

### 2.1 Eligibility Criteria

The “PECO” framework guiding the definition of the eligibility criteria was as follows:

- P (population): patients with Apert or Crouzon syndrome;
- E (exposure): dental evaluation of patients for the presence of permanent tooth agenesis;
- C (comparison): no comparison group included, as prevalence evaluated without a direct comparison;
- O (outcome): prevalence and patterns of permanent tooth agenesis.

Consequently, the predefined inclusion criteria were: human studies; any observational study design (case series or cross‐sectional or longitudinal studies, retrospective or prospective); individuals diagnosed with Apert or Crouzon syndrome, without restrictions on age, sex, ethnicity, or country of origin; studies looking at the prevalence and/or patterns of tooth agenesis either clinically and/or radiographically.

The exclusion criteria were: animal studies; the inclusion of individuals with syndromic craniosynostosis other than Apert or Crouzon syndrome or with non‐syndromic craniosynostosis; case reports; and the inclusion of teeth absent due to caries or periodontal disease as opposed solely to permanent tooth agenesis.

### 2.2 Information Sources

A literature search was conducted to identify studies pertaining to permanent tooth agenesis in individuals with Apert or Crouzon syndrome with the last search being carried out in June 2025, using MEDLINE (via PubMed), Web of Science, Embase, ProQuest (Dissertations and Theses), and Google Scholar.

### 2.3 Search Strategy

A comprehensive search strategy was designed using combinations of Medical Subject Headings (MeSH), Emtree terms (for Embase), and relevant free‐text terms, including truncations (e.g., hypodont*). The search strategy on PubMed used the following terms: (“Apert Syndrome”[MeSH] OR “Crouzon Syndrome”[MeSH] OR “Craniosynostoses”[MeSH] OR Apert or Crouzon OR craniosynostosis) AND (“Tooth Abnormalities”[MeSH] OR “Anodontia”[MeSH] OR “Oligodontia”[MeSH] OR “Congenital Absence of Teeth” OR “Agenesis of Permanent Teeth” OR hypodontia OR anodontia OR oligodontia OR “dental agenesis” OR “tooth agenesis” OR “missing teeth” OR “congenitally missing teeth” OR hypodont* OR agenesi*). To retrieve additional articles, the “related citations” tool in PubMed was utilised alongside citation tracking methods. Initial retrieval of studies was unrestricted regarding publication date, language, or status. Reference lists of selected articles were reviewed manually, and searches were conducted for prominent authors in the field to ensure comprehensive coverage of relevant literature. Search strategies used on the other searched registries were built in a similar logic but adapted to each database's index syntax. The results from Google Scholar, sorted by relevance, were screened using the keywords “Apert syndrome” AND “dental agenesis”, “Apert syndrome” AND “tooth agenesis”, “Crouzon syndrome” AND “dental agenesis”, “Crouzon syndrome” AND “tooth agenesis”. The complete search strategies are provided in Table S1.

### 2.4 Selection Process

Two reviewers carried out the literature search independently and manually screened the titles and abstracts initially retrieved. Duplicates were also removed manually. Full‐text articles were retrieved for all potentially eligible records or unclear records based on their title and abstract and were assessed for eligibility using the predefined criteria. Any disagreements were resolved through discussion and consultation with a third reviewer to reach a consensus on which articles should be included.

### 2.5 Data Collection Process and Data Items

Data extraction was conducted independently by two reviewers, and the extracted data from the selected studies were cross‐verified for accuracy. Any disagreements between the reviewers were resolved through discussion with a third reviewer. Extracted information included publication data (journal and year of publication, authors), study and sample characteristics (study type, sample size, age, sex distribution, diagnosis—Apert and/or Crouzon syndrome, ethnic origin of included individuals), outcome characteristics and data (how tooth agenesis was determined and by how many examiners, error of the method assessment, overall and individual tooth agenesis prevalence, patterns of tooth agenesis).

When patterns of permanent tooth agenesis were identified or inferred, the Tooth Agenesis Code (TAC) [18] was used to record these observations. This coding system employs binary notation to represent tooth agenesis in each dental quadrant, where 0 represents the presence of a tooth and 1 indicates its absence. Each missing tooth is assigned a specific value based on the formula 2^(*n*−1)^, with *n* corresponding to the tooth's position, ranging from 1 (central incisor) to 8 (third molar), following the FDI (Fédération Dentaire Internationale) notation. These values are summed within each dental quadrant, producing a unique numerical representation of permanent tooth agenesis for that quadrant. A quadrant without any tooth agenesis receives a TAC value of 0, while one with complete agenesis is assigned a value of 255. For the entire dentition, the TAC overall aggregates the quadrant‐specific values into a single notation in the format TACq1.TACq2.TACq3.TACq4, where q1 through q4 represent the first through fourth quadrants. This system provides a comprehensive and standardised method to describe the distribution of permanent tooth agenesis across the dentition.

### 2.6 Study Risk of Bias Assessment

The quality assessment of the included studies was performed independently by two reviewers, and the tool used was the Joana Briggs Institute (JBI) critical appraisal checklist for studies reporting prevalence data [19]. Any disagreements between reviewers were resolved by consulting a third reviewer.

### 2.7 Synthesis Methods and Effect Measures

All statistical analyses were performed using MetaXL version 2.0 (www.epigear.com). Given the variability in participant age and origin, a random‐effects meta‐analysis model was applied to account for heterogeneity. Additionally, the inverse variance heterogeneity model was also employed [20]. The inverse variance heterogeneity model addresses limitations of the random‐effects model by preventing underestimation of statistical error and overly optimistic estimates.

As in a previous study [10], prevalence rates were transformed using the double arcsine method. Since meta‐analyses of prevalence can disproportionately emphasise studies with values at the extremes of the 0–1 range, this double arcsine transformation has been recommended to resolve issues with confidence intervals exceeding the 0–1 range and to stabilise variance. Accordingly, in each meta‐analysis model used, the effect size represented the prevalence of tooth agenesis, but the data were analysed using the double arcsine transformation. For clarity, the final results were reverted to proportions for presentation, including 95% confidence intervals (95% CI).

Heterogeneity was evaluated using Cochran's *Q* test [21] and the *I* ^2^ statistic [22]. Sensitivity analyses were conducted by systematically excluding individual studies to observe any changes in the final estimates. Forest plots were used to present the pooled prevalence estimates and subgroup comparisons.

Publication bias and reporting bias were not assessed using funnel plots or Egger's test, due to an insufficient number of included studies. Similarly, the certainty of evidence was not formally evaluated using the GRADE approach due to the limited number and observational nature of the studies included.

## 3 Results

### 3.1 Study Selection

**FIGURE 1:**
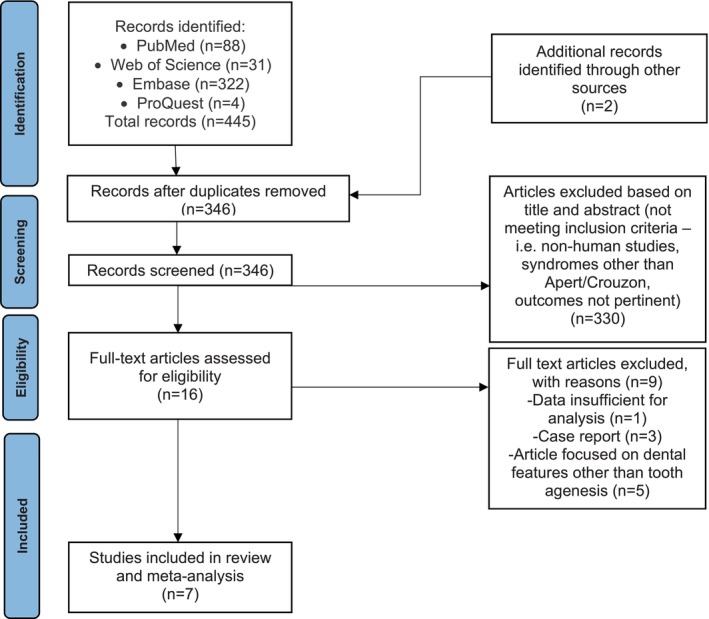
PRISMA flow diagram illustrating the identification, full screening, eligibility, and inclusion process of studies in the systematic review and meta‐analysis.

The initial literature search identified a total of 445 articles. After the initial deduplication, 346 articles were screened, including 2 additional articles identified through other sources. Of these, 16 were sought for full‐text retrieval and were thoroughly assessed for eligibility. Following this evaluation, 7 articles met the inclusion criteria and were included in the present study (Figure 1) [9, 23, 24, 25, 26, 27, 28]. The authors of three articles were contacted for additional data [9, 24, 25], two of whom sent the data requested [9, 25].

### 3.2 Study Characteristics and Results of Individual Studies

The characteristics of the seven included studies are presented in Table 1. These studies were published between 2006 and 2021. The final sample comprised 89 individuals with Apert syndrome and 77 patients with Crouzon syndrome, with a total of 166 individuals, ranging from roughly 5 to 22 years of age. The sample originated from four countries (namely Brazil, Sweden, the Netherlands, and Japan), in three continents, and five centres within these countries.

**TABLE 1:** Characteristics of the studies included in the systematic review and meta‐analysis.

Authors	Year	Country	Syndrome (sample size)	Age (years)	Sex distribution	Prevalence of tooth agenesis
Dalben et al. [26]	2006	Brazil	Apert (*n* = 9)	Range = 6–15	N/R	44.4%
Letra et al. [24]	2007	Brazil	Apert (*n* = 17)	Mean = 17.7	N/R	41.2%
Stravropoulos et al. [23]	2011	Sweden	Apert (*n* = 23)	Inclusion ≥ 8	5 males 18 females	34.8%
Stravropoulos et al. [27]	2011	Sweden	Crouzon (*n* = 26)	Inclusion ≥ 8	20 males 6 females	42.3%
Reitsma et al. [9]	2014	The Netherlands	Apert (*n* = 28)	Range = 11–22	10 males 18 females	28.6%
			Crouzon (*n* = 39)	Range = 11–22	20 males 19 females	20.5%
Kakutani et al. [28]	2017	Japan	Apert (*n* = 5)	Range = 5–9	3 males 2 females	40.0%
Kobayashi et al. [25]	2021	Japan	Apert (*n* = 7)	Mean = 12.3 ± 5	4 males 3 females	57.1%
			Crouzon (*n* = 12)	Mean = 10.8 ± 2.9	6 males 6 females	33.3%

The overall prevalence rates of permanent tooth agenesis ranged from 28.6% to 57.1% in individuals with Apert syndrome and from 20.5% to 42.3% in individuals with Crouzon syndrome. In only six of the individual studies was it possible to ascertain specific permanent tooth agenesis patterns [9, 23, 25, 26, 27, 28]. In those with permanent tooth agenesis, the number of missing teeth ranged from 1 to 5 in both groups with Apert and Crouzon syndromes.

### 3.3 Risk of Bias in Studies

The risk of bias in the included studies is summarised in Table S2. Overall, the included studies demonstrated a moderate to high risk of bias. While the domains related to sample frame and sampling methods were adequately addressed, the rest of the domains were generally deemed either unclear or of high risk.

### 3.4 Meta‐Analysis

#### 3.4.1 Overall Prevalence

**FIGURE 2:**
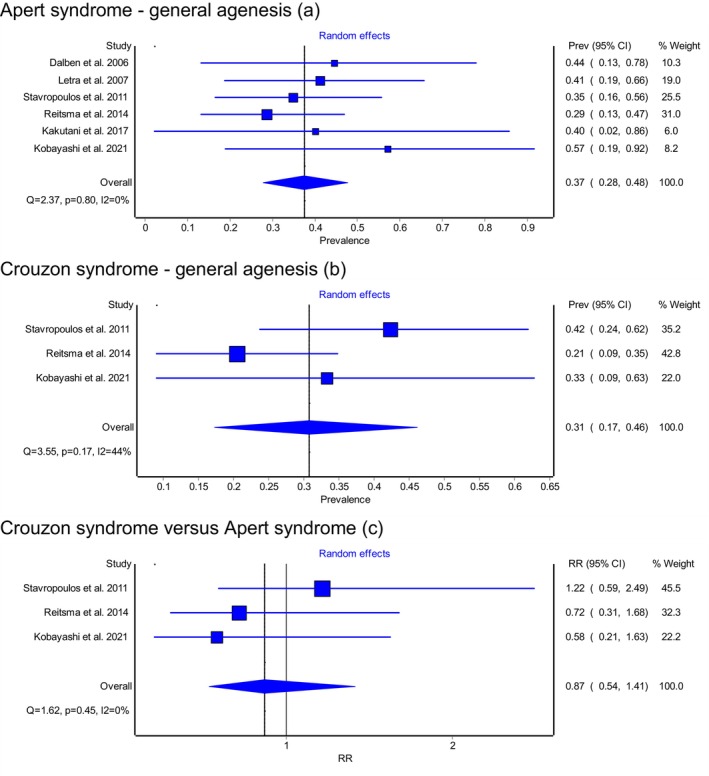
General agenesis observed in patients with (a) Apert syndrome, (b) Crouzon syndrome, and (c) comparing Crouzon to Apert syndrome, using the random‐effects meta‐analysis model. 95% CI = 95% confidence interval; RR = risk ratio.

Based on the meta‐analyses conducted, the estimated prevalence of permanent tooth agenesis (excluding third molars) was 37% (95% CI: 28%–48%) in individuals with Apert syndrome and 31% (95% CI: 17%–46%) in those with Crouzon syndrome, both derived using the random‐effects model (Figure 2). The inverse variance heterogeneity model did not show substantially different results from the random‐effects model, and thus only the results of the random‐effects meta‐analyses will be presented for the sake of brevity.

A direct comparison between the two syndromes using the random‐effects model (with low heterogeneity, *I* ^2^ = 0%) yielded a pooled risk ratio (RR) of 0.87 (95% CI: 0.54–1.41), indicating that patients with Crouzon syndrome have a 13% lower risk of tooth agenesis than those with Apert syndrome, although this difference was not statistically significant (Figure 2). No sex differences were found regarding the prevalence of permanent tooth agenesis in either syndrome.

#### 3.4.2 Comparison Between Left and Right Sides, and Maxilla and Mandible

When assessing the distribution of tooth agenesis, meta‐analyses of relative risk revealed no statistically significant differences between left‐ and right‐sided agenesis or between maxillary and mandibular agenesis.

#### 3.4.3 Unilateral Versus Bilateral Tooth Agenesis

Meta‐analyses of relative risk showed no statistically significant differences between unilateral and bilateral tooth agenesis for either syndrome (Figure S1). This finding supports a relatively symmetrical distribution of tooth agenesis in both conditions.

### 3.5 Monomaxillary Versus Bimaxillary Tooth Agenesis

A further comparison between monomaxillary and bimaxillary tooth agenesis demonstrated a significant difference in Apert syndrome, with an RR of 2.67 (95% CI: 1.08–6.60), suggesting a potential trend towards higher prevalence of monomaxillary agenesis (Figure S1). In Crouzon syndrome, the corresponding RR was 2.28 (95% CI: 0.99–5.23), which suggests a similar trend but without reaching statistical significance. Given the small sample size, the proximity of the RR to 1.0, and the wide confidence interval, this result should be interpreted with caution (Figure S1).

Comparisons of single‐tooth versus multiple‐tooth agenesis in both syndromes revealed no significant differences (Figure S1).

#### 3.5.1 Tooth Agenesis by Tooth Type

Prevalence estimates of agenesis of permanent tooth types in individuals with Apert syndrome, based on the random‐effects meta‐analyses model, are shown in Table 2. These estimates were derived from five [9, 23, 25, 26, 28] studies that provided sufficient data, with a total sample of 89 individuals with Apert syndrome. Similarly, for Crouzon syndrome, the prevalence estimates were obtained from 3 [9, 25, 27] studies, including a total of 77 patients (Table 2). The permanent teeth most frequently affected by agenesis in individuals with Apert syndrome, excluding third molars, were the mandibular second premolars and the maxillary lateral incisors. In contrast, for individuals with Crouzon syndrome, the most affected teeth were the mandibular second premolars, followed by the maxillary second premolars. Heterogeneity was low and largely insignificant, and sensitivity analyses confirmed the consistency of these results.

**TABLE 2:** Estimates of agenesis prevalence of permanent tooth types with the most frequent agenesis, using two different meta‐analysis models, with heterogeneity data also presented. 95% CI = 95% confidence interval; prev = prevalence. Percentages are calculated based exclusively on patients with tooth agenesis.

	Maxillary teeth					
	Lateral incisor		Second premolar		Second molar	
	Prev	95% CI	Prev	95% CI	Prev	95% CI
Apert syndrome						
Random‐effects model	18%	5%–36%	4%	1%–11%	2%	0%–7%
Inverse variance heterogeneity model	15%	2%–32%	4%	1%–11%	2%	0%–7%
Heterogeneity						
Cochran's *Q*	10.0 (*p* = 0.04)		3.6 (*p* = 0.47)		0.8 (*p* = 0.94)	
*I* squared	60		0		0	
Crouzon syndrome						
Random‐effects model	6%	1%–14%	10%	4%–17%	7%	0%–22%
Inverse variance heterogeneity model	5%	1%–13%	10%	4%–17%	5%	0%–20%
Heterogeneity						
Cochran's *Q*	2.6 (*p* = 0.27)		1.8 (*p* = 0.40)		8.0 (*p* = 0.02)	
*I* squared	23				75	

	Mandibular teeth			
	Central or lateral incisors		Second premolar	
	Prev	95% CI	Prev	95% CI
Apert syndrome				
Random‐effects model	2%	0%–6%	17%	9%–27%
Inverse variance heterogeneity model	2%	0%–6%	17%	9%–27%
Heterogeneity				
Cochran's *Q*	3.6 (*p* = 0.47)		3.4 (*p* = 0.49)	
*I* squared	0		0	
Crouzon syndrome				
Random‐effects model	7%	2%–14%	15%	8%–24%
Inverse variance heterogeneity model	7%	2%–14%	15%	8%–24%
Heterogeneity				
Cochran's *Q*	1.1 (*p* = 0.57)		1.2 (*p* = 0.56)	
*I* squared	0		0	

#### 3.5.2 Tooth‐Specific Prevalence

**FIGURE 3:**
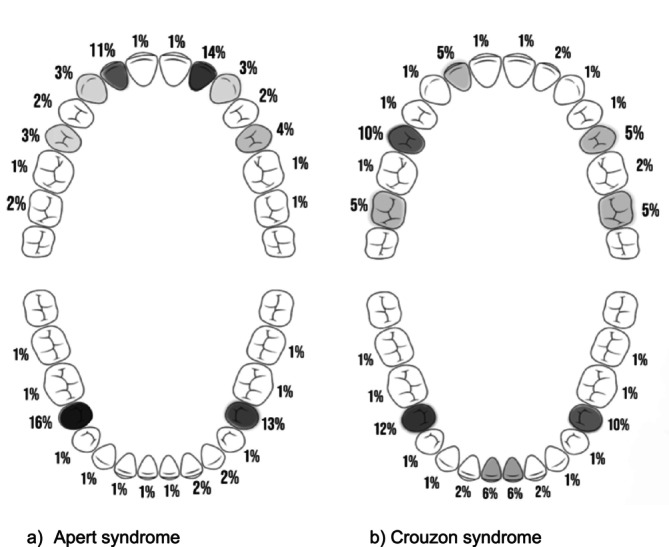
Prevalence of permanent tooth agenesis of specific teeth in individuals with Apert syndrome (a) and Crouzon syndrome (b), based on the random‐effects metaanalysis model (teeth with highest, high, medium and low relative prevalence of agenesis are highlighted in black, dark grey, light grey and white, respectively).

The estimated prevalence of agenesis for individual permanent teeth in Apert syndrome ranged from 1%–16%, while for Crouzon syndrome, it ranged from 1%–12%, based on the random‐effects meta‐analysis model. These overall estimates were derived from 89 individuals with Apert syndrome across five studies and from 77 individuals with Crouzon syndrome across three studies. In Apert syndrome, the permanent teeth with the highest prevalence of agenesis, excluding third molars, were the right mandibular second premolar (16%), left maxillary lateral incisor (14%), left mandibular second premolar (13%), and right maxillary lateral incisor (11%) (Figure 3). In Crouzon syndrome, the most affected teeth were the right mandibular second premolar (12%), right maxillary second premolar (10%), left mandibular second premolar (10%), and mandibular right and left central incisors (6% each) (Figure 3).

#### 3.5.3 Permanent Tooth Agenesis Patterns

**FIGURE 4:**
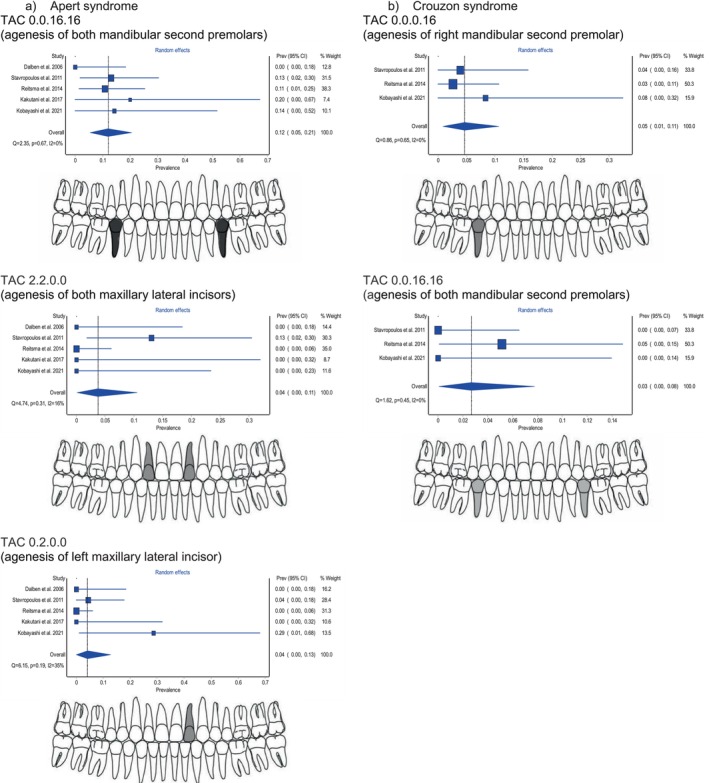
Forest plots presenting the estimated prevalence of the most common tooth agenesis patterns in individuals with Apert syndrome (a) and Crouzon syndrome (b), using the random‐effects meta‐analysis model. The shaded odontograms illustrate the most frequent tooth agenesis patterns identified across studies (using the Tooth Agenesis Code – TAC). Each TAC pattern represents a quadrant‐based binary code indicating missing teeth in the following order: upper right, upper left, lower left, and lower right consistent with the FDI World Dental Federation notation.

Furthermore, specific permanent tooth agenesis patterns were analysed by converting the available data from the five Apert syndrome studies (Table S3a) and the three Crouzon syndrome studies (Table S3b) into a TAC overall score. In Apert syndrome, the most frequent pattern was 0.0.16.16, representing the bilateral absence of the mandibular second premolars (Figure 4). This was followed by the pattern 0.2.0.0, indicating the absence of the left maxillary lateral incisor, and 2.2.0.0, denoting the bilateral absence of the maxillary lateral incisors. In Crouzon syndrome, the most common pattern was 0.0.0.16, indicating the absence of the right mandibular second premolar, followed by 0.0.16.16, which represents the absence of both mandibular second premolars (Figure 4).

## 4 Discussion

The present meta‐analysis provides a comprehensive estimate of the prevalence and distribution patterns of permanent tooth agenesis in individuals with Apert syndrome and Crouzon syndrome. The overall prevalence of permanent tooth agenesis, excluding third molars, was 37% in individuals with Apert syndrome and 31% in individuals with Crouzon. The pooled RR suggests a 13% lower risk of permanent tooth agenesis in Crouzon compared to Apert syndrome, though this difference was not statistically significant. No sex‐related differences in permanent tooth agenesis were observed in either syndrome.

Regarding the distribution of agenesis, a relatively symmetrical pattern was identified, with no significant differences between left‐ and right‐sided agenesis, maxillary versus mandibular agenesis, or unilateral versus bilateral agenesis. However, a significant difference was observed in the occurrence of monomaxillary versus bimaxillary agenesis in Apert syndrome (RR = 2.67, 95% CI: 1.08–6.60), suggesting a higher prevalence of monomaxillary agenesis. A similar trend was found in Crouzon syndrome (RR = 2.28, 95% CI: 0.99–5.23), though it did not reach statistical significance.

The most commonly missing tooth in both syndromes was the right mandibular second premolar, absent in 16% of individuals with Apert and 12% of individuals with Crouzon syndrome. The most prevalent permanent tooth agenesis pattern in Apert syndrome was the bilateral absence of the mandibular second premolars (prevalence estimate of 12%; observed in 7 out of 26 individuals with permanent tooth agenesis), while in Crouzon syndrome, the most common pattern was the absence of the right mandibular second premolar (prevalence estimate of 5%; observed in 3 out of 23 individuals with permanent tooth agenesis).

In healthy populations, meta‐analyses show that permanent tooth agenesis occurs in 3.2%–7.6% of individuals [1], compared with an overall prevalence of 28%–48% in Apert syndrome and 17%–46% in Crouzon syndrome in the present study. Although the data are not directly comparable, this represents roughly an estimated sevenfold increase in Apert syndrome and a sixfold increase in Crouzon syndrome.

The mandibular second premolar is the most common tooth affected by tooth agenesis in both the general population and in individuals with Apert syndrome or Crouzon syndrome. However, prevalence rates vary significantly, with estimates of 17% for Apert syndrome and 15% for Crouzon syndrome in this study, compared to 2.9%–3.2% in a healthy population [1]. These results emphasise that while tooth agenesis is a common feature in both syndromes, the specific prevalence rates suggest that the genetic mechanisms involved may differentially affect dental development in Apert and Crouzon syndromes.

The findings of this analysis are consistent with previous studies reporting a high prevalence of permanent tooth agenesis in both syndromes. Dalben et al. [26] and Letra et al. [24] identified the mandibular second premolars and maxillary lateral incisors as the most frequently missing teeth in Apert syndrome, which aligns with these results. Similarly, Stavropoulos et al. [27] reported that agenesis of the second premolars is predominant in Crouzon syndrome, a trend also supported here. However, the significant monomaxillary agenesis pattern in Apert syndrome had not been emphasised before, suggesting that further research is needed to explore this finding.

These results have important clinical implications. Missing premolars and lateral incisors may compromise arch form development and occlusion, particularly in patients who already present maxillary hypoplasia, midface retrusion, and/or class III skeletal patterns. This further complicates long‐term orthodontic planning.

From a clinical perspective, the management of patients with Apert or Crouzon syndrome must be a multidisciplinary and coordinated approach. Common dental complications in these patients include delayed eruption, ectopic tooth positioning, severe crowding, and difficulties in maintaining adequate oral hygiene. Early involvement of paediatric dentists, orthodontists, and oral and maxillofacial surgeons is essential to guide the timing of interventions and minimise complications [29].

In syndromic patients, tooth agenesis can represent a significant challenge for both orthodontic and prosthodontic management. Early diagnosis of these patterns is crucial for implementing interceptive measures, including space maintenance if needed, consideration of mini‐implant placement during orthodontic treatment, prosthetic planning, and appropriate sequencing of surgical interventions.

Despite the significance of these findings, certain limitations must be acknowledged. A key concern is the lack of complete information in several studies, including essential details such as patient age, sex, or detailed data on missing teeth, which could affect the accuracy of prevalence estimates and the identification of specific agenesis patterns. Additionally, variability in sample sizes, diagnostic methods, and inclusion criteria contributes to heterogeneity across studies.

Methodological inconsistencies within individual studies, such as the use of a single examiner or uncalibrated examiners, a lack of inter‐rater reliability data, and differences in diagnostic thresholds, may have influenced the pooled prevalence estimates. Notably, some studies included participants at ages when dental mineralisation, especially of the second premolars, may not yet be complete, particularly in syndromic patients with genetic and signalling pathway alterations that may delay or disrupt normal odontogenesis, increasing the risk of misdiagnosing delayed development as agenesis. These factors may lead to either underestimation or overestimation of true prevalence rates.

Additionally, although ethnicity was not an exclusion criterion, the included studies involved populations with diverse ethnic and genetic backgrounds. In individuals diagnosed with either Apert or Crouzon syndrome, phenotypic variability in the expression of tooth agenesis may be influenced by additional genetic modifiers, gene–environment interactions, or epigenetic regulation. These factors could partly explain the variability in prevalence rates and tooth agenesis patterns observed across studies.

Another important challenge in studying Apert and Crouzon syndromes is the rarity of these syndromes. Recruiting large numbers of patients for more precise and comprehensive studies is inherently difficult. While future research with standardised diagnostic protocols would be ideal, the feasibility of conducting large‐scale studies remains limited due to the low prevalence of these conditions. Alternative approaches, such as international collaborations and centralised databases, could help address this challenge and provide more robust data on tooth agenesis in these populations.

Given these sources of heterogeneity, the findings of this meta‐analysis contribute meaningfully to the field; however, they reflect the diversity and complexity inherent in the included data sources and should be interpreted as exploratory in nature. Further studies investigating tooth agenesis in individuals with Apert or Crouzon syndrome are necessary to refine the impact of tooth agenesis on long‐term orthodontic and prosthodontic outcomes to optimise treatment strategies and multidisciplinary management. Future research should also incorporate detailed genetic analyses to investigate the molecular mechanisms underlying the aetiopathogenesis of the syndrome and the subsequent phenotypic expressions, including tooth agenesis, potentially leading to early diagnostic markers and targeted therapeutic interventions. Evidence suggests that dental development in these syndromes follows a characteristic pattern influenced by mutations in the *FGFR2* and *FGFR3* genes [14, 16]. These mutations lead to gene inactivation, interrupting tooth development at the bud stage, as these genes play a crucial role in cell proliferation [12, 15].

### 4.1 Conclusions

The present study indicates that individuals with Apert or Crouzon syndrome have a high prevalence of permanent tooth agenesis (excluding third molars), with estimated prevalence rates ranging from 28%–48% in Apert syndrome and 17%–46% in Crouzon syndrome. The most frequently affected teeth in Apert syndrome were the mandibular second premolars and maxillary lateral incisors, while in Crouzon syndrome, the mandibular second premolars were the most affected, followed by the maxillary second premolars. Further research associating permanent tooth agenesis with genetic analyses may help in identifying early diagnostic markers and developing targeted therapeutic interventions.

## Author Contributions

**M. Cecilia Becerril Santos:** methodology, data extraction, data analysis, drafting of the manuscript, and final approval of the version to be published. **Edwin M. Ongkosuwito:** methodology, critical revision of the manuscript, and final approval of the version to be published. **Alexandra K. Papadopoulou:** supervision, methodology, critical revision of the manuscript, final approval of the version to be published. **Gregory S. Antonarakis:** conceptualisation, supervision, methodology, data extraction, critical revision of the manuscript, final approval of the version to be published.

## Ethics Statement

The authors have nothing to report.

## Consent

The authors have nothing to report.

## Conflicts of Interest

The authors declare no conflicts of interest.

## Supporting information


**Data S1:** ocr70046‐sup‐0001‐Supplementary Material.docx.

## Data Availability

All data generated or analysed during this study are included in this article and its Supporting Information files.
