# Supplementary material for: Prevalence and Patterns of Permanent Tooth Agenesis in Patients With Crouzon or Apert Syndrome: A Systematic Review and Meta‐Analysis
Source: Orthod Craniofac Res. 2025 Oct 14;29(1):1–11. doi: 10.1111/ocr.70046 (PMC12779208; doi:10.1111/ocr.70046)
Supplement: Supplementary file 1 — Data S1: ocr70046‐sup‐0001‐Supplementary Material.docx. [file OCR-29-1-s001.docx]

SUPPLEMENTARY MATERIAL

| DATABASE | SEARCH STRATEGY |
| --- | --- |
| PubMed | ("Apert Syndrome"[MeSH] OR "Crouzon Syndrome"[MeSH] OR "Craniosynostoses"[MeSH] OR apert OR crouzon OR craniosynostosis) AND ("Tooth Abnormalities"[MeSH] OR "Anodontia"[MeSH] OR "Oligodontia"[MeSH] OR "Congenital Absence of Teeth" OR "Agenesis of Permanent Teeth" OR hypodontia OR anodontia OR oligodontia OR "dental agenesis" OR "tooth agenesis" OR "missing teeth" OR "congenitally missing teeth" OR hypodont* OR agenesi*) |
| Embase | (‘apert syndrome’/exp OR ‘crouzon syndrome’/exp OR ‘craniosynostosis’/exp OR apert OR crouzon OR craniosynostosis) AND (‘tooth anomaly’/exp OR ‘anodontia’/exp OR ‘oligodontia’/exp OR ‘tooth agenesis’ OR ‘dental agenesis’ OR ‘congenital tooth absence’ OR hypodontia OR hypodont* OR agenesi*) |
| Web of Science | (TS=(apert OR crouzon OR craniosynostosis)) AND (TS=(“tooth agenesis” OR “dental agenesis” OR “missing teeth” OR hypodontia OR hypodont* OR agenesi* OR oligodontia OR anodontia OR “congenitally missing teeth” OR “congenital absence of teeth”)) |
| ProQuest | TI,AB(apert OR crouzon OR craniosynostosis) AND TI,AB(“tooth agenesis” OR “dental agenesis” OR hypodontia OR hypodont* OR agenesi* OR oligodontia OR anodontia OR “congenitally missing teeth” OR “missing teeth”) |
| Google scholar | “Apert syndrome” AND “dental agenesis”, “Apert syndrome” AND “tooth agenesis”, “Crouzon syndrome” AND “dental agenesis”, “Crouzon syndrome” AND “tooth agenesis” |

Supplementary Table S1. Search strategies by database (PubMed, Embase, Web of Science, Proquest and Google scholar).

| First author and year | Sample frame | Sampling method | Sample size calculation | Subjects and  setting | Coverage of data analysis | Identifications methods | Condition measurement | Statistical analysis | Response rate | TOTAL |
| --- | --- | --- | --- | --- | --- | --- | --- | --- | --- | --- |
| Dalben et al. 2006 |  |  |  |  |  |  |  |  |  |  |
| Letra et al. 2007 |  |  |  |  |  |  |  |  |  |  |
| Stravropoulos et al. 2011 |  |  |  |  |  |  |  |  |  |  |
| Stravropoulos et al. 2011 |  |  |  |  |  |  |  |  |  |  |
| Reitsma et al. 2014 |  |  |  |  |  |  |  |  |  |  |
| Kakutani et la. 2017 |  |  |  |  |  |  |  |  |  |  |
| Kobayashi et al. 2021 |  |  |  |  |  |  |  |  |  |  |

Supplementary Table S2: Risk of bias assessment using the JBI tool. Red colour indicates high risk of bias, green indicates low risk of bias and yellow indicates unclear risk of bias.

1. Crouzon syndrome

| TAC | Teeth missing (using FDI notation) | Number of missing teeth | Total number of individuals | Prevalence estimate (mean; 95% CI) (%) |
| --- | --- | --- | --- | --- |
| 0.0.0.16 | 45 | 1 | 3 | 0.05 (0.01-0.11) |
| 0.0.16.16 | 35,45 | 2 | 2 | 0.02 (0.00-0.08) |
| 0.0.16.0 | 35 | 1 | 1 | 0.02 (0.00-0.06) |
| 16.0.0.0 | 15 | 1 | 1 | 0.02 (0.00-0.06) |
| 2.0.0.0 | 12 | 1 | 1 | 0.02 (0.00-0.06) |
| 0.2.0.0 | 22 | 1 | 1 | 0.02 (0.00-0.06) |
| 64.0.0.0 | 17 | 1 | 1 | 0.02 (0.00-0.06) |
| 0.64.0.0 | 27 | 1 | 1 | 0.02 (0.00-0.06) |
| 0.32.0.0 | 16 | 1 | 1 | 0.02 (0.00-0.06) |
| 16.0.0.16 | 15,45 | 2 | 1 | 0.02 (0.00-0.06) |
| 64.64.0.0 | 17,27 | 2 | 1 | 0.02 (0.00-0.06) |
| 0.0.2.2 | 32,42 | 2 | 1 | 0.02 (0.00-0.06) |
| 16.0.16.16 | 15,35,45 | 3 | 1 | 0.02 (0.00-0.06) |
| 18.16.0.0 | 15,12,25 | 3 | 1 | 0.02 (0.00-0.06) |
| 2.0.16.16 | 12,35,45 | 3 | 1 | 0.02 (0.00-0.06) |
| 16.0.1.1 | 15,31,41 | 3 | 1 | 0.02 (0.00-0.06) |
| 0.1.1.1 | 21,31,41 | 3 | 1 | 0.02 (0.00-0.06) |
| 0.0.17.1 | 35,31,41 | 3 | 1 | 0.02 (0.00-0.06) |
| 16.80.16.16 | 15,25,27,35,45 | 5 | 1 | 0.02 (0.00-0.06) |
| 80.16.1.1 | 17,15,25,31,41 | 5 | 1 | 0.02 (0.00-0.06) |

b) Apert syndrome

| TAC | Teeth missing (using FDI notation) | Number of missing teeth | Total number of individuals | Prevalence estimate (mean; 95% CI) (%) |
| --- | --- | --- | --- | --- |
| 0.0.16.16 | 35,45 | 2 | 7 | 0.12 (0.05-0.21) |
| 2.2.0.0 | 12,22 | 2 | 3 | 0.04 (0.00-0.11) |
| 0.2.0.0 | 22 | 1 | 3 | 0.04 (0.00-0.13) |
| 2.0.0.0 | 12 | 1 | 1 | 0.02 (0.00-0.06) |
| 0.16.0.0 | 25 | 1 | 1 | 0.02 (0.00-0.07) |
| 0.0.16.0 | 35 | 1 | 1 | 0.02 (0.00-0.07) |
| 0.0.0.16 | 45 | 1 | 1 | 0.02 (0.00-0.06) |
| 4.0.0.0 | 13 | 1 | 1 | 0.02 (0.00-0.06) |
| 0.4.0.0 | 23 | 1 | 1 | 0.02 (0.00-0.06) |
| 8.8.0.0 | 14,24 | 2 | 1 | 0.02 (0.00-0.07) |
| 64.0.0.16 | 17,45 | 2 | 1 | 0.02 (0.00-0.07) |
| 2.2.2.0 | 12,22,32 | 3 | 1 | 0.02 (0.00-0.06) |
| 2.0.16.16 | 12,35,45 | 3 | 1 | 0.02 (0.00-0.07) |
| 16.18.0.0 | 15,22,25 | 3 | 1 | 0.02 (0.00-0.06) |
| 16.16.16.16 | 15,25,35,45 | 4 | 1 | 0.02 (0.00-0.07) |
| 6.6.0.4 | 13,12,22,23,33 | 5 | 1 | 0.02 (0.00-0.06) |

Supplementary Table S3: Tooth agenesis pattern in a) a sample of 23 patients with Crouzon syndrome and permanent tooth agenesis, from a total sample of 72 patients derived from 3 studies; b) a sample of 26 patients with Apert syndrome and permanent tooth agenesis, from a total sample of 77 patients derived from 3 studies

1. left- versus right-sided tooth agenesis

Apert syndrome Crouzon syndrome

1. unilateral versus bilateral tooth agenesis

Apert syndrome Crouzon syndrome

1. monomaxillary versus bimaxillary tooth agenesis

Apert syndrome Crouzon syndrome

1. single-tooth versus multiple tooth agenesis

Apert syndrome Crouzon syndrome

Supplementary Figure S1. Forest plots presenting the estimated relative risk with regard to permanent tooth agenesis in individuals with Apert syndrome or Crouzon syndromes: (a) left- versus right-sided tooth agenesis; (b) unilateral versus bilateral tooth agenesis; (c) monomaxillary versus bimaxillary tooth agenesis; (d) single-tooth vs multiple tooth agenesis.
